# Trigeminal neuralgia secondary to multiple sclerosis: from the clinical picture to the treatment options

**DOI:** 10.1186/s10194-019-0969-0

**Published:** 2019-02-19

**Authors:** Giulia Di Stefano, Stine Maarbjerg, Andrea Truini

**Affiliations:** 1 Department of Human Neurosciences, Sapienza University, Viale Università 30, 00185 Rome, Italy; 2 Danish Headache Center, Department of Neurology, Rigshospitalet - Glostrup, University of Copenhagen, Copenhagen, Denmark

**Keywords:** Secondary trigeminal neuralgia, Multiple sclerosis, Neuropathic pain

## Abstract

**Background:**

Trigeminal neuralgia is one of the most characteristic and difficult to treat neuropathic pain conditions in patients with multiple sclerosis. The present narrative review addresses the current evidence on diagnostic tests and treatment of trigeminal neuralgia secondary to multiple sclerosis.

**Methods:**

We searched for relevant papers within PubMed, EMBASE and the Cochrane Database of Systematic Reviews, taking into account publications up to December 2018.

**Results:**

Trigeminal neuralgia secondary to multiple sclerosis manifests with facial paroxysmal pain triggered by typical manoeuvres; neurophysiological investigations and MRI support the diagnosis, providing the definite evidence of trigeminal pathway damage. A dedicated MRI is required to identify pontine demyelinating plaques. In many patients with multiple sclerosis, neuroimaging and surgical evidence suggests that neurovascular compression might act in concert with the pontine plaque through a double-crush mechanism. Although no placebo-controlled trials have been conducted in these patients, according to expert opinion the first-line therapy for trigeminal neuralgia secondary to multiple sclerosis relies on sodium-channel blockers, i.e. carbamazepine and oxcarbazepine. The sedative and motor side effects of these drugs frequently warrant an early consideration for neurosurgery. Surgical procedures include Gasserian ganglion percutaneous techniques, gamma knife radiosurgery and microvascular decompression in the posterior fossa.

**Conclusions:**

The relatively poor tolerability of the centrally-acting drugs carbamazepine and oxcarbazepine highlights the need to develop new selective and better-tolerated sodium-channel blockers. Prospective studies based on more advanced neuroimaging techniques should focus on how trigeminal anatomical abnormalities may be able to predict the efficacy of microvascular decompression.

## Background

Multiple sclerosis (MS) is a chronic inflammatory disease causing demyelination and axonal degeneration in the central nervous system. Neuropathic pain is a common symptom in patients with MS. Among the different types of neuropathic pain, trigeminal neuralgia (TN) is a characteristic and difficult to treat neuropathic pain condition, with a relevant impact on the quality of life [1]. Patients with MS experiencing TN find that daily life activities, work, mood, recreation and overall quality of life can be disrupted [1].

In this narrative review, we aim at addressing the current evidence on TN secondary to MS.

## Methods

We searched relevant papers within the PubMed, EMBASE and the Cochrane Database of Systematic Reviews, taking into account publications up to December 2018. All searches used the following keywords: multiple sclerosis AND trigeminal neuralgia, multiple sclerosis AND facial pain. The primary search was supplemented by a secondary search using the bibliographies of the articles retrieved. Only full-length, original communications were accepted, and the search was limited to English language publications. This search yielded a total of approximately 400 articles, which were reviewed by title and abstract for potential relevance to this topic; when the title and abstract did not clearly indicate the degree of relevance to the topic, the article itself was reviewed.

## Results

### Definitions and epidemiology

The International Classification of Headache Disorders [2] and the TN classification issued by the Special Interest Group on Neuropathic Pain of the International Association for the Study of Pain distinguish between classical TN, caused by a vascular compression producing morphological changes in the trigeminal nerve root, secondary TN, which is due to an identifiable underlying neurological disease, and idiopathic TN [3]. In patients with idiopathic TN even advanced diagnostic investigations fail to show a cause. TN is characterized by recurrent, unilateral, brief, electric shock-like pain, abrupt in onset and termination. Pain is limited to the distribution of one or more divisions of the trigeminal nerve and triggered by innocuous stimuli. Additionally, there may be concomitant continuous pain of moderate intensity within the distribution(s) of the affected nerve division(s). Secondary TN occurs in up to 15% [4–6] of TN patients and the diagnosis is made in the presence of a structural abnormality affecting the trigeminal nerve other than vascular compression, including multiple sclerosis (MS) plaques, tumours and abnormalities of the skull base. MS plaques are the most commonly identified abnormalities. Patients with MS have a 20-fold increased risk of developing TN [7]; 1.9–4.9% of patients with MS suffer from this neuropathic pain condition [8–12], without differences between relapsing-remitting, secondary and primary progressive forms [8]; conversely MS is detected in 2%–14% of patients with TN [10].

### Clinical characteristics

TN secondary to MS is, like the classical and idiopathic TN, characterized by a sudden, usually unilateral, brief, stabbing or electrical shock-like, recurrent pain with a distribution that is consistent with one or more divisions of the fifth cranial nerve. The paroxysmal attacks, last from a fraction of a second to 2 min and are typically evoked by stimulating cutaneous or mucous trigeminal territories, i.e. the so-called trigger zones. Gently touching the face, washing, shaving, talking, tooth brushing, chewing, swallowing or even a slight breeze may trigger the paroxysms. Stimulus-dependence is considered one of the most striking characteristics of TN and a criterion of clinically-established TN [3]. Patients may also report spontaneous attacks. However, it is still an issue of controversy whether these pain attacks are elicited by very subtle sensory stimuli or movements or are genuine spontaneous attacks [6]. The frequency of the pain attacks may range from 1 to over 50 a day [4, 13]. Patients with classical and idiopathic TN have pain-free intervals of often complete remission lasting from weeks to years, most often a few months [6]. Conversely, there is a lack of general consensus about the occurrence of remission periods in TN secondary to MS. Remission periods are probably due to a reduction in excitability and partial remyelination, but evidence is missing to support this hypothesis [14]. These pain characteristics are easily differentiated from other MS-related neuropathic facial pain conditions, including ongoing pain, dysesthesias and provoked pain. Some patients with TN secondary to MS, as well as patients with classical and idiopathic TN, suffer from concomitant continuous, dull, burning, or tingling pain between the paroxysms. The distribution of continuous pain coincides with that of the paroxysmal pain, and fluctuations in intensity as well as periods of remission and recurrence parallel those of the paroxysmal pain [6].

TN secondary to MS is, like classical and idiopathic TN, more common in women than in men, and affects the right side more frequently than the left side [15, 16]. TN secondary to MS tends, however, to occur at an earlier age in patients with MS, with age at onset ranging from 40 to 50 years [15, 16]. The first branch alone may be involved in TN secondary to MS, though the second and/or the third branch are involved in approximately 90% of cases [5, 6, 15]. Although the characteristics of TN secondary to MS are similar to those observed in classical TN, the pain is more frequently bilateral in MS patients, with an estimated 18% of patients reported to have bilateral TN [15–17]. Clinical deficits of discriminatory sensory functions, which are highly indicative of secondary TN, occur in 37% of patients with secondary TN [5, 8]. Although a younger age and trigeminal sensory deficits are associated with an increased risk of secondary TN and should be considered useful for distinguishing secondary TN from classical TN, the absence of these clinical features does not rule out TN secondary to MS [7, 18].

### Pathophysiological mechanisms

**Fig. 1:**
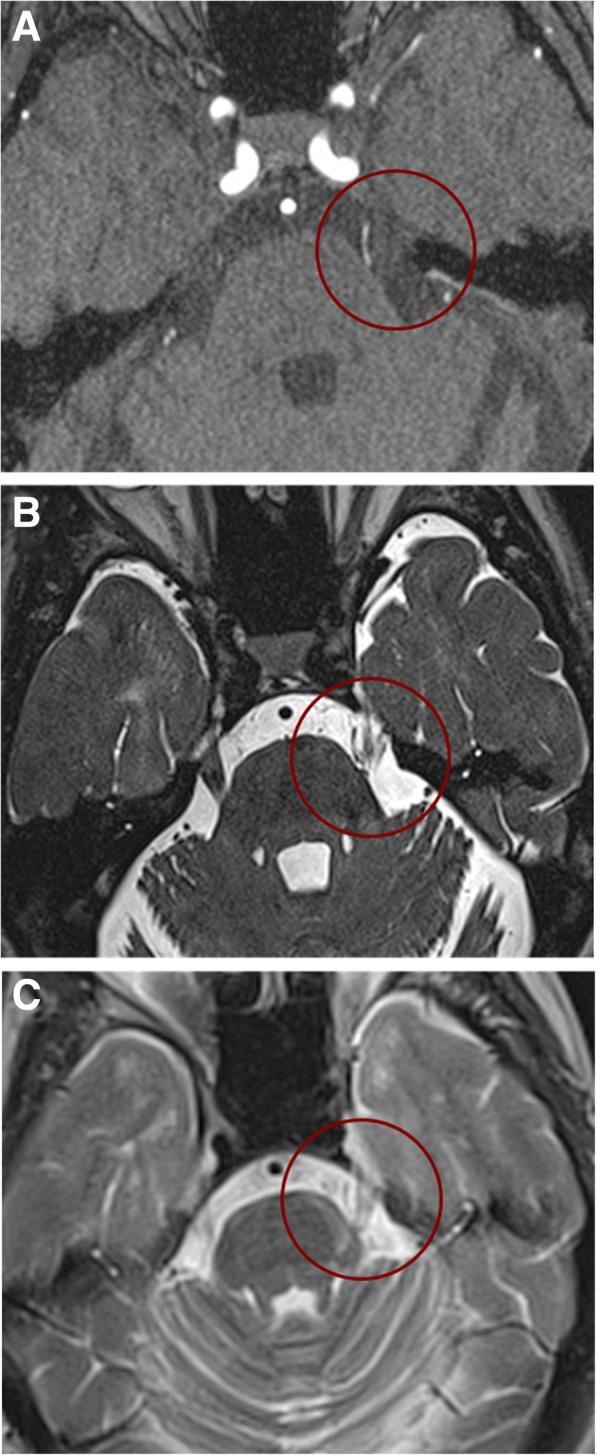
Neuroimaging findings in a representative patient with TN secondary to MS possibly due to a double crush mechanism. 3D time-of-flight (TOF) magnetic resonance angiography scans (**a**) and 3D constructive interference in the steady state (CISS) T2-weighted (**b**) on the axial plane demonstrate left neurovascular compression (NVC) with associated trigeminal nerve atrophy. T2-weighted image on the axial plane shows a hyperintense pontine lesion at the left trigeminal nerve root entry zone (REZ) (**c**). The arrow indicates the trigeminal nerve (**b**) and the arrowhead the demyelinating plaque (**c**). Reproduced from [16]

Established knowledge postulates that TN secondary to MS is associated with a pontine demyelinating plaque. A neurophysiological and neuroimaging study in patients with TN secondary to MS showed that the lesion involves the anatomical area corresponding to the intrapontine segment of the trigeminal nerve, an area centred in the ventrolateral pons between the trigeminal root entry zone (REZ) and the trigeminal nuclei, i.e. along the intrapontine trigeminal primary afferents [15]. The role of the pontine demyelinating plaque is also supported by functional neuroimaging studies showing that tensor imaging abnormalities in patients with classical and idiopathic TN are located in the cisternal and REZ segments of the trigeminal nerve, whereas in patients with TN secondary to MS the abnormalities are located in the pontine tract of the trigeminal nerve [19]. Although TN secondary to MS has long been attributed exclusively to a demyelinating plaque affecting the trigeminal REZ in the pons [15, 20, 21], the plaque theory contrasts with the frequent neuroimaging findings of neurovascular compression of the trigeminal root in patients with TN secondary to MS and with the observation in some MS patients that TN is the sole clinical manifestation (Fig. 1) [22]. A prospective clinical and neuroimaging study in patients with MS revealed a significant association between neurovascular compression and TN secondary to MS, thus suggesting that a pontine plaque affecting the intra-axial primary afferents and neurovascular compression in concert might cause TN secondary to MS through a double-crush mechanism, involving inflammatory demyelination and mechanical demyelination, of the same first-order neurons [16].

There is broad consensus that the primary mechanism of paroxysmal pain in TN is the focal demyelination of primary afferents at the entry of the trigeminal root into the pons. This area represents a locus of minoris resistentiae since it is here that Schwann cells are replaced by oligodendroglia to form the myelin sheath [23]. As a result of demyelination, the axons tend toward a depolarization level, which makes them hyperexcitable. This, in turn, produces ectopic excitation, high-frequency discharges and ephaptic transmission from neighbouring, healthy nerve fibres [24–26]. A possible secondary effect of the hyperactivity of primary afferents is central sensitization of wide-dynamic-range neurons in the trigeminal spinal nucleus or changes that are even more central, but more research is needed into these pathophysiological mechanisms [27].

Focal demyelination is not the only mechanism underlying the development of paroxysmal pain in patients with TN. The immediate pain relief following microvascular decompression cannot be explained by a remyelination process, thus suggesting a possible role of a transient conduction block. This hypothesis was supported by the immediate recovery of trigeminal root conduction, demonstrated by both scalp evoked potentials and direct root recordings, after microvascular decompression [28].

### Diagnostic tests

According to the classification and diagnostic grading system for practice and research issued by the Special Interest Group on Neuropathic Pain of the International Association for the Study of Pain [3], the diagnosis of secondary TN relies on the demonstration of a major neurologic disease that damages the trigeminal pathway and causes neuralgia. In patients with TN secondary to MS, neurophysiological techniques and MRI are commonly used to provide a definite evidence of trigeminal pathway impairment [18]. Although various neurophysiological techniques can be used to assess the trigeminal system, trigeminal reflex testing has a diagnostic specificity and sensitivity close to 90% for identifying trigeminal pathway impairment in patients with secondary TN [5]. This technique is easier and less invasive than the evoked-potential technique, with the finding of any abnormality suggesting an underlying structural lesion. The trigeminal reflexes consist of a series of reflex responses (R1 and R2 components of the blink reflex after electrical stimulation of the ophthalmic division, SP1 and SP2 components of the masseter inhibitory reflex after electrical stimulation of the maxillary or mandibular division) that assess the functioning of the trigeminal afferents from all trigeminal territories, as well as the trigeminal central circuits in the midbrain, pons and medulla [29]. Trigeminal reflex testing is abnormal in 89% of patients with TN secondary to MS but in only 3% of patients with classical and idiopathic TN [5]. In patients without a relevant pontine plaque and with normal trigeminal reflex testing, one can speculate whether it is theoretically plausible that classical or idiopathic TN can co-exist with MS in one and the same patient.

**Fig. 2:**
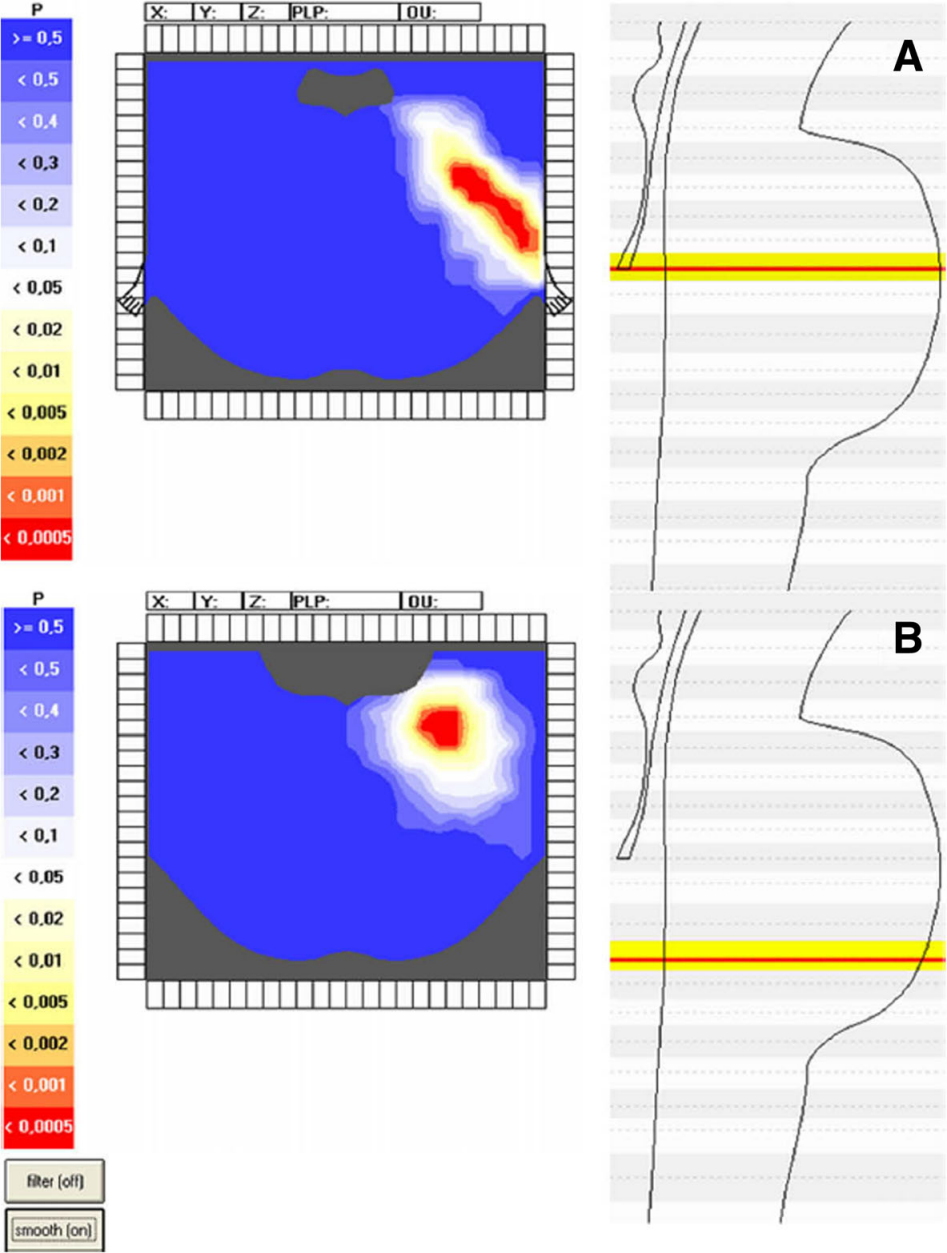
Voxel-based analysis in patients with TN secondary to MS. Voxel-based brainstem model in patients with TN secondary to MS (TN group, *n* = 42) and in patients with trigeminal sensory disturbances due to MS (non-TN group, *n* = 29). The statistical analysis in patients with TN secondary to MS showed an area of very high probability of a lesion (*P* < 0.0001) centred in the ventrolateral pons between the trigeminal root entry zone and the trigeminal nuclei, i.e. along the intrapontine part of the trigeminal primary afferents. In the non-TN group, the area of high probability of lesion (*P* < 0.001) corresponded to a more caudal, medial, and dorsal pontine region involving the subnucleus oralis of the spinal trigeminal complex. The axial sections in this figure correspond to the sections 120 and 160 of the Shaltenbrandt atlas. The level of probability is colour-coded. Blue indicates non-significant areas, white the minimum level of significance (*P* < 0.05), and red the highest level of significance. Reproduced from [15]

MRI is routinely used for diagnosing MS and identifying TN secondary to MS. In patients with TN secondary to MS, T2-weighted MRI scans identify any linear plaques in the ventrolateral pons located between the trigeminal root entry zone and the trigeminal nuclei and involving the intrapontine part of primary afferents of the trigeminal nerve [15, 30]. Conversely, brainstem lesions in patients with MS-related trigeminal sensory disturbances other than TN (ongoing pain, dysesthesia or hypoesthesia) are more scattered, with lesions most likely to be found in the region involving the subnucleus oralis of the spinal trigeminal complex (Fig. 2) [15].

Since MRI can be used to reliably investigate the anatomy and vascular relationships of the trigeminal nerve, it is useful for assessing the neurovascular compression of the trigeminal nerve at the root entry zone. Previous studies showed that neurovascular compression, i.e. with morphological changes of the trigeminal nerve such as atrophy, dislocation, indentation or flattening, was highly associated with the symptomatic side in TN patients with MS [16, 31]. This finding indicates a more complex disease aetiology with at least two causes of demyelination in some TN patients with MS.

Admittedly, the MRI identification of a pontine plaque in patients with confirmed MS do not probably influence treatment strategies. Conversely, the MRI investigation of the neurovascular conflict may be important for planning microvascular decompression as surgical treatment.

### Treatment

#### Pharmacological treatment

Pharmacological treatment of TN secondary to MS is challenging owing to the poor tolerability of drugs and the lack of evidence-based information in the literature. There are no placebo-controlled studies, and the studies that do exist are small, open-label trials based on carbamazepine (CBZ), lamotrigine, gabapentin, topiramate, misoprostol or combination therapies [32–43]. These case series reported potential efficacy of lamotrigine as monotherapy or associated with gabapentin or carbamazepine, topiramate and gabapentin. Pregabalin was tested in a pilot study investigating the effect on painful paroxysmal symptoms in sixteen patients with MS, including two patients with TN [35]. Lamotrigine, with a mean dosage of 170 mg daily, significantly reduced pain related to TN in a group of 18 patients with MS [33]. In a recent, prospective, open-label, pilot study five patients with TN secondary to MS were successfully treated by a combination treatment of pregabalin plus lamotrigine [43]. The effect of topiramate was tested in six patients with MS and TN refractory to conventional medical therapy. Five out of six patients treated with topiramate (50–300 mg/day) reported complete pain relief [36]. Three studies reported efficacy of misoprostol (a prostaglandin-E1-analogue) in a total of 28 patients with TN secondary to MS [37, 44, 45]. Reder and Arnason reported that misoprostol (300–800 μg) relieved pain in six of seven patients who had failed to respond to conventional pharmacologic therapy, without serious side effects [37]. DMKG study group tested the effect of misoprostol (600 μg) in refractory TN associated with MS. Eighteen patients completed the study period and 14 of them showed a reduction of more than 50% in attack frequency and intensity beginning five days after treatment onset. There were only mild and transient drug-related side effects in three patients [45]. According to the international guidelines [18], there is insufficient evidence to support or refute the effectiveness of any medication in treating pain in TN secondary to MS. It is, however, generally agreed that the first line therapy is pharmacological and is based, as it is for classical and idiopathic TN, on the use of sodium-channel blockers, i.e. CBZ and oxcarbazepine (OXC) [46, 47]. These drugs block voltage-gated sodium-channels in a frequency-dependent manner and consequently reduce their action-potential firing frequency. Placebo-controlled trials in patients with classical and idiopathic TN demonstrated the efficacy of CBZ [48, 49], with a number needed to treat to obtain important pain relief of 1.7–1.8 [50]. However, this efficacy in classical and idiopathic TN is compromised by the tolerability, with a number needed to harm of 3.4 for minor adverse events and of 24 for severe adverse events [51, 52]. The most frequent adverse effects involve the central nervous system, and include somnolence, dizziness and postural imbalance. OXC has a comparable efficacy to that of CBZ but a greater tolerability [53] (except of the risk of hyponatremia) and a lower potential for drug interaction [54, 55]. In TN secondary to MS, many patients never advance to the regimen required for pain relief because of intolerable adverse effects. CBZ and OXC can result in adverse effects that mimic a disease exacerbation, and the sudden onset or sudden worsening of common MS symptoms may, consequently, be erroneously treated with intravenous steroids [32, 56]. As in classical and idiopathic TN, these drugs may be combined with lamotrigine, baclofen, pregabalin or gabapentin in patients that are unable to attain a full dosage of CBZ or OXC because of side effects [57].

Patients suffering from persistent pain between the paroxysms are more resistant to CBZ and OXC [57]. These drugs produce a frequency-dependent block of voltage-gated sodium channels and, thereby, by reducing the frequency of action potential firing, they effectively reduce paroxysmal pain; however, they have a far less positive effect on concomitant persistent pain. According to clinical experience, gabapentinoids and antidepressants might be more effective in persistent than in paroxysmal pain and are often tried as an add-on to OXC or CBZ in patients with the atypical form of TN with concomitant persistent pain [57]. No trial, however, has directly assessed the efficacy of this combination in patients with persistent pain and there is no evidence to support or refute its use in clinical practice [57].

A recent phase 2A study investigated the efficacy of a novel selective sodium-channel 1.7 blocker in patients with classical TN [58]. This novel drug, which targets nociceptive sodium-channel afferents and has no effect on the CNS, is likely to be tolerated better than CBZ and OXC.

#### Surgical treatments

Although the role of surgery in the management of TN secondary to MS remains uncertain [18], it is generally agreed that patients who do not respond to or cannot attain the therapeutic dosage required should be informed of the availability of surgery [22, 59, 60]. Reported outcomes on case series of TN patients with MS indicate that surgical procedures in such patients tend to be less effective than in patients with classical and idiopathic TN [61–63]. The majority of available neurosurgical studies, however, are retrospective and without independent assessors of outcome.

A reduced long-term benefit in comparison with patients with classical and idiopathic TN and the occurrence of potentially serious adverse events suggest that surgical procedures should be reserved for medically refractory patients. Several authors have suggested that continuous pain in patients with TN is associated with poorer outcome after surgical intervention [47, 51] but this conclusion is still controversial. Surgical procedures include peripheral lesions distal to the ganglion, gasserian ganglion percutaneous techniques, stereotactic radiosurgery and microvascular decompression in the posterior fossa [64–66]. The first group of surgical methods includes peripheral lesions of the trigeminal terminal nerves at their emergence from the facial bones: neurectomy, alcohol injections, radiofrequency lesions, or cryolesions. These procedures are usually well tolerated but none of these methods has ever been supported by adequate trials [67].

Percutaneous ganglion lesions include thermocoagulation by radiofrequency, chemical lesions by injection of high-concentration glycerol and mechanical compression by balloon inflation. Even though results vary in different case series, no convincing superiority of any surgical method has emerged in this patient category [68]. The major risks of all percutaneous ganglion lesion procedures are piercing of the maxillary artery and that of the dura mater covering the Meckel cave, with various possible consequences, from burning of an oculomotor nerve to infusion of glycerol into the CSF of the middle cranial fossa. Trigeminal sensory deficits are almost unavoidable; these are usually transient with balloon compression and glycerol injection and more severe and longer lasting after radiofrequency [68, 69].

Several studies with a follow-up exceeding one year have investigated the role of surgical procedures designed to lesion the Gasserian ganglion. Procedures were performed chemically by glycerol injections [61, 70–72], mechanically by balloon compression [73–76], or thermically by radiofrequency thermocoagulation [64, 77–79]. Although most patients enrolled in these studies reported complete acute pain relief following the lesioning procedures, the recurrence rate during follow-up and the frequency of adverse events varied widely (Table 1). In the case series by Mohammad-Mohammadi and colleagues, a total of 96 patients underwent 277 procedures to treat TN secondary to MS, including percutaneous glycerol injection, balloon compressions, stereotactic radiosurgery, radiofrequency thermocoagulation and microvascular decompression. Symptoms recurred in 66% of patients and 181 procedures were performed for symptom recurrence. As an initial procedure, balloon compression had the highest initial pain-free response and median pain-free intervals, followed by glycerol injection [59]. There are no significant differences in the frequency of complications associated with the lesioning procedures. Each patient should thus be thoroughly informed of the advantages and limitations of each procedure, so that the most appropriate one can be chosen with the surgeon as an alternative option for the treatment of TN secondary to MS.

**Table 1:** Summary of studies dealing with gangliolysis techniques and gamma knife radiosurgery in patients with multiple sclerosis-related trigeminal neuralgia

Gasserian ganglion percutaneous techniques						
Author	Procedure	no	Complete pain relief* (%)	Mean follow-up (months)	Complication rate (%)	Recurrence rate (%)
Broggi, 1982	Radiofrequency rhizotomy	14	100	NA	NA	40
Hooge and Redekop, 1995	Radiofrequency rhizotomy	17	57	72	NA	43
Kanpolat, 2000	Radiofrequency rhizotomy	17	70,6	60	76,5	29,4
Berk, 2003	Radiofrequency rhizotomy	13	81	52	0	50
Mallory, 2012	Radiofrequency rhizotomy	67	40	28.3	3	54
Holland, 2017	Radiofrequency rhizotomy	10	71	66	66	60
Dieckmann, 1987**	Glycerol rhizotomy	21	NA	NA	NA	40
Kondziolka, 1994	Glycerol rhizotomy	53	60	36	0	40
Pickett, 2005	Glycerol rhizotomy	53	78	81	20	59
Mathieu, 2012	Glycerol rhizotomy	18	100	38	66,7	38.9
Mohammad-Mohammadi, 2013	Glycerol rhizotomy	39	74	68,4	3	69
Kouzounias, 2010	Balloon compression	17	88	20	0	70,5
Mallory, 2012	Balloon compression	69	26	17.8	17.4	64
Montano, 2012	Balloon compression	21	81	51,5	0	57
Mohammad-Mohammadi, 2013	Balloon compression	19	95	68,4	5	61
Bergenheim, 2013**	Balloon compression	23	NA	NA	NA	NA
Martin, 2015	Balloon compression	17	82	43	21	86
Alvarez-Pinzon, 2016	Balloon compression	78	87	18	21	NA
Rogers, 2002	Stereotactic radiosurgery	15	80	17	13	33.3
Zorro, 2009	Stereotactic radiosurgery	37	62.1	56.7	5.4	37.8
Verheul, 2010	Stereotactic radiosurgery	13	90	16	37	35
Mathieu, 2012	Stereotactic radiosurgery	27	89	39	22.2	51.9
Weller, 2014	Stereotactic radiosurgery	35	35	39	39	40.7
Tuleasca, 2014	Stereotactic radiosurgery	43	90.7	53.8	16	61.5
Alvarez-Pinzon, 2016	Stereotactic radiosurgery	124	23	24	10	NA
Holland, 2017	Stereotactic radiosurgery	7	71	10	60	29
Conti, 2017	Stereotactic radiosurgery	27	85	37	26	56
Colin, 2018	Stereotactic radiosurgery	42	62	78	10	87
Broggi et al., 2004	Microvascular decompression	35	39	44	NA	NA
Athanasiou et al., 2005	Microvascular decompression	5	100	38.8	0	20
Eldridge et al., 2003	Microvascular decompression	9	100	12	11	66
Sandell T and Eide, 2010	Microvascular decompression	15	47	65	33	NA
Ariai et al., 2014	Microvascular decompression	10	80	14	10	60

NA not available

*Pain relief with no need of pharmacological treatment

**These studies investigated patients with classical and MS-related TN and does not provide distinct data of the two conditions

Other studies with a follow-up exceeding one year have investigated the role of stereotactic radiosurgery in patients with TN secondary to MS [65, 80–83]. The probability of remaining pain-free without resorting to medication in five years and the frequency of adverse events are still unclear. In one case series of TN patients with MS who underwent stereotactic radiosurgery, only 38% of the patients were still pain-free without drugs after five years. The frequency of complications, which consist of trigeminal sensory disturbances was ranging widely from 5 to 57% [84]. A recent retrospective review of long-term outcomes involving 42 patients showed that the proportion of patients with pain relief after stereotactic radiosurgery was 62%, 29%, 22%, and 13% at 1, 3, 5, and 7 years [85]. Unlike the other types of intervention, the pain-relieving effect of stereotactic radiosurgery is not immediate and generally requires 6 to 8 weeks to develop. Another issue is the reliability and accuracy of the methods of finding the exact coordinates of the trigeminal root just before its entrance into the pons, where the radiation beams should collimate. On the other hand, stereotactic radiosurgery is associated with a lower rate of adverse events than Gasserian ganglion procedures. These two techniques may be therefore considered as valuable alternatives for treating TN secondary to MS, with the choice between them being based on the patient’s and surgeon’s preferences. Retrospective studies have compared the efficacy of stereotactic radiosurgery and Gasserian ganglion procedures [70, 86]. These studies have shown that patients treated with Gasserian ganglion procedures experience immediate pain relief and do not need to resort to TN drugs for longer periods than patients treated with stereotactic radiosurgery. In a recent study involving a small sample of patients radiofrequency thermocoagulation and stereotactic radiosurgery provide initial pain relief in 71% of patients. Over time, 60% of radiofrequency thermocoagulation and 29% of stereotactic radiosurgery patients required additional procedures to obtain satisfactory pain relief [87].

The conventional opinion that MS is an absolute contraindication to microvascular decompression, due to the supposed exclusive causative role of a demyelinating lesion affecting the trigeminal root entry zone, has been contrasted by some studies supporting the role of vascular compression in MS patients [22, 88, 89]. Neurovascular compression may act as a concurring mechanism that leads to the focal demyelination of primary afferents near the entry of the trigeminal root into the pons. This hypothesis is supported by the fact that severe neurovascular compression at the trigeminal root entry zone is found in most patients during surgery (from 50% to 100% of patients with TN secondary to MS) [20, 90, 91]. Microvascular decompression in patients with classical TN produces immediate pain relief in the majority of patients. However, when applied to patients with TN secondary to MS, this technique is generally reported to be less effective than in patients with classical TN. Indeed, after five years fewer than 50% of the patients in the case series described by Broggi and 15% in the case series described by Ariai were still pain-free (in comparison with approximately 80% of pain-free patients after surgery for classical TN). The adverse event rate of microvascular decompression is very low. In the two aforementioned case series, only one patient suffered long-term morbidity (facial nerve palsy). In larger case series by Barker et al. on microvascular decompression in patients with classical TN, the rate of adverse events was also low but included death (0.2%), brainstem infarction (0.1%), cerebellar hematoma and edema (0.5%) and severe or permanent cranial nerve damage (3%). This major surgical procedure requires general anesthesia, intubation and craniotomy. Given the serious nature of some of the reported adverse events, thorough presurgical patient information is important [92].

The reduced efficacy of microvascular decompression in TN secondary to MS points to the crucial role of the pontine demyelinating plaque in most patients with this form of TN; however, the observation that this surgical procedure is still effective in many patients lends support to the involvement of neurovascular compression in TN secondary to MS. On the other hand, we cannot exclude that, during microvascular decompression, manipulation of the trigeminal root may be a sufficiently traumatic procedure to disrupt the parossistic discharge behaviour of the axons. Before drawing definitive conclusions, we must await further high-quality evidence demonstrating that microvascular decompression is indeed an effective technique. Further studies using advanced neuroimaging techniques like diffusion tensor imaging (DTI) and 3.0 Tesla MRI are also warranted. Possibly, such studies have the potential to identify the trigeminal anatomical abnormalities that can predict the outcome of the different neurosurgical procedures and thereby guide future clinical decision-making and patient information.

## Conclusions and future perspectives

Patients with MS suffer from various types of neuropathic pain, the most severe being TN, which has a significant impact on quality of life [1]. The relatively poor tolerability of the sedative and motor side effects of the centrally-acting drugs CBZ and OXC highlight the need to develop new selective and better-tolerated sodium-channel blockers. A new selective sodium-channel 1.7 blocker is under development [58].

Although there is evidence demonstrating that neurovascular compression may act as a concurring mechanism in some patients with TN secondary to MS, we still lack high-quality research assessing the efficacy of microvascular decompression in MS patients. Hence, prospective studies using independent assessors of outcome and advanced neuroimaging techniques should focus on how trigeminal anatomical abnormalities may be able to predict the efficacy of microvascular decompression.

## Abbreviations

CBZ: carbamazepine
DTI: diffusion tensor imaging
MS: multiple sclerosis
OXC: oxcarbazepine
REZ: root entry zone
TN: trigeminal neuralgia
